# A mitochondrial function-related LncRNA signature predicts prognosis and immune microenvironment for breast cancer

**DOI:** 10.1038/s41598-023-30927-y

**Published:** 2023-03-08

**Authors:** Yuan Wang, Shun Gao, Yingkun Xu, Zhenrong Tang, Shengchun Liu

**Affiliations:** Department of Breast and Thyroid Surgery, The First Affiliated Hospital of Chongqing Medical University, Chongqing, China

**Keywords:** Cancer, Computational biology and bioinformatics, Biomarkers

## Abstract

Mitochondrial function, as the core of the cell's energy metabolism, is firmly connected to cancer metabolism and growth. However, the involvement of long noncoding RNAs (lncRNAs) related to mitochondrial function in breast cancer (BRCA) has not been thoroughly investigated. As a result, the objective of this research was to dissect the prognostic implication of mitochondrial function-related lncRNAs and their link to the immunological microenvironment in BRCA. The Cancer Genome Atlas (TCGA) database was used to acquire clinicopathological and transcriptome information for BRCA samples. Mitochondrial function-related lncRNAs were recognized by coexpression analysis of 944 mitochondrial function-related mRNAs obtained from the MitoMiner 4.0 database. A novel prognostic signature was built in the training cohort using integrated analysis of mitochondrial function-related lncRNA and the corresponding clinical information through univariate analysis, lasso regression, and stepwise multivariate Cox regression analysis. The prognostic worth was judged in the training cohort and validated in the test cohort. In addition, functional enrichment and immune microenvironment analyses were performed to explore the risk score on the basis of the prognostic signature. An 8-mitochondrial function-related lncRNA signature was generated by integrated analysis. Individuals within the higher-risk category had a worse overall survival rate (OS) (training cohort: P < 0.001; validation cohort: P < 0.001; whole cohort: P < 0.001). The risk score was identified as an independent risk factor by multivariate Cox regression analysis (training cohort: HR 1.441, 95% CI 1.229–1.689, P < 0.001; validation cohort: HR 1.343, 95% CI 1.166–1.548, P < 0.001; whole cohort: HR 1.241, 95% CI 1.156–1.333, P < 0.001). Following that, the predictive accuracy of the model was confirmed by the ROC curves. In addition, nomograms were generated, and the calibration curves revealed that the model had excellent prediction accuracy for 3- and 5-year OS. Besides, the higher-risk BRCA individuals have relatively decreased amounts of infiltration of tumor-killing immune cells, lower levels of immune checkpoint molecules, and immune function. We constructed and verified a novel mitochondrial function-related lncRNA signature that might accurately predict the outcome of BRCA, play an essential role in immunotherapy, and might be exploited as a therapeutic target for precise BRCA therapy.

## Introduction

As the most common malignant tumor among women and one of the chief causes of cancer death, breast cancer (BRCA) has a prognosis that is far from satisfactory despite the availability of comprehensive therapy options such as surgery, radiation, chemotherapy, and immunotherapy^1–3^. Therefore, it is critical to gain better insight into new prognostic biomarkers to prognosticate and screen the high-risk population with a worse prognosis, advance new therapeutic approaches, and provide timely targeted intervention for BRCA.

Mitochondria are involved in multiple functions in tumors, such as genomic regulation, metabolic control, and adaptive immune responses^4,5^. Academic interest in the mitochondrial function of cancer cells has gradually increased in recent decades. The malignant phenotypes of tumor cells are also closely connected with abnormal mitochondrial function, such as infinite proliferation, abnormal metabolism, resistance to apoptosis, invasion, and metastasis^6^. The in-depth study on the influences of the abnormalities of mitochondrial function-related genes in BRCA is of profound significance for understanding the process of tumor genesis and development as well as the prognosis of BRCA. Therefore, it is essential to identify mitochondrial function-related biomarkers that can serve as prognostic indicators for BRCA.

Long non-coding RNAs (lncRNAs) are critical in the genesis and advancement of malignancy and might symbolize therapeutic marks or possible indicators^7^. In recent years, researchers have even noticed that lncRNAs could be more precise than other forms of indicators in determining the status of the tumor^8^. Although some previous studies focused on mitochondrial dysfunction in tumor cells, there are limited studies on mitochondrial function-related lncRNAs in BRCA, and a large number of lncRNAs that regulate mitochondrial function have not been fully understood. High-throughput sequencing is a fast-growing technique that may provide a basis for uncovering mitochondrial function-related and prospective prognostic biomarkers. Therefore, we conducted this integrated bioinformatics analysis to investigate the function of mitochondrial function-associated lncRNAs and explore their potential value in forecasting survival for BRCA.

Mitochondria are integral to the regulation of immune function, and dysfunctional mitochondria are involved in a variety of procedures that are closely associated with abnormalities in the immune system^9,10^. However, there is limited published literature that focuses on the association between mitochondrial function and tumor immunity in BRCA. Therefore, it was of interest to investigate the link between mitochondrial function-related lncRNAs and the BRCA tumor immune microenvironment (TIM) as well as immunotherapy.

The purpose of this study was to identify mitochondrial function-related lncRNAs in BRCA that could provide valuable insight into the molecular and signaling pathways of mitochondrial function, as well as function as biomarkers to predict the survival of BRCA. In summary, we constructed a mitochondrial function-related lncRNA signature, which presents good predictive accuracy for predicting the outcome and may provide a theoretical foundation for targets of immunological therapy in BRCA.

## Methods

### Data acquisition

The fragments per kilobase of per million (FPKM) of RNA-seq transcriptome profiling, and clinical data from BRCA were retrieved from the Cancer Genome Atlas (TCGA) database. The study complies with the TCGA Data Access Policy and Publication Guidelines for all data sources that are available to the public. The ensembl human genome browser GRCh38.p13 was used to distinguish the protein-coding genes and lncRNAs and identify lncRNA expression^11^.

**Figure 1:**
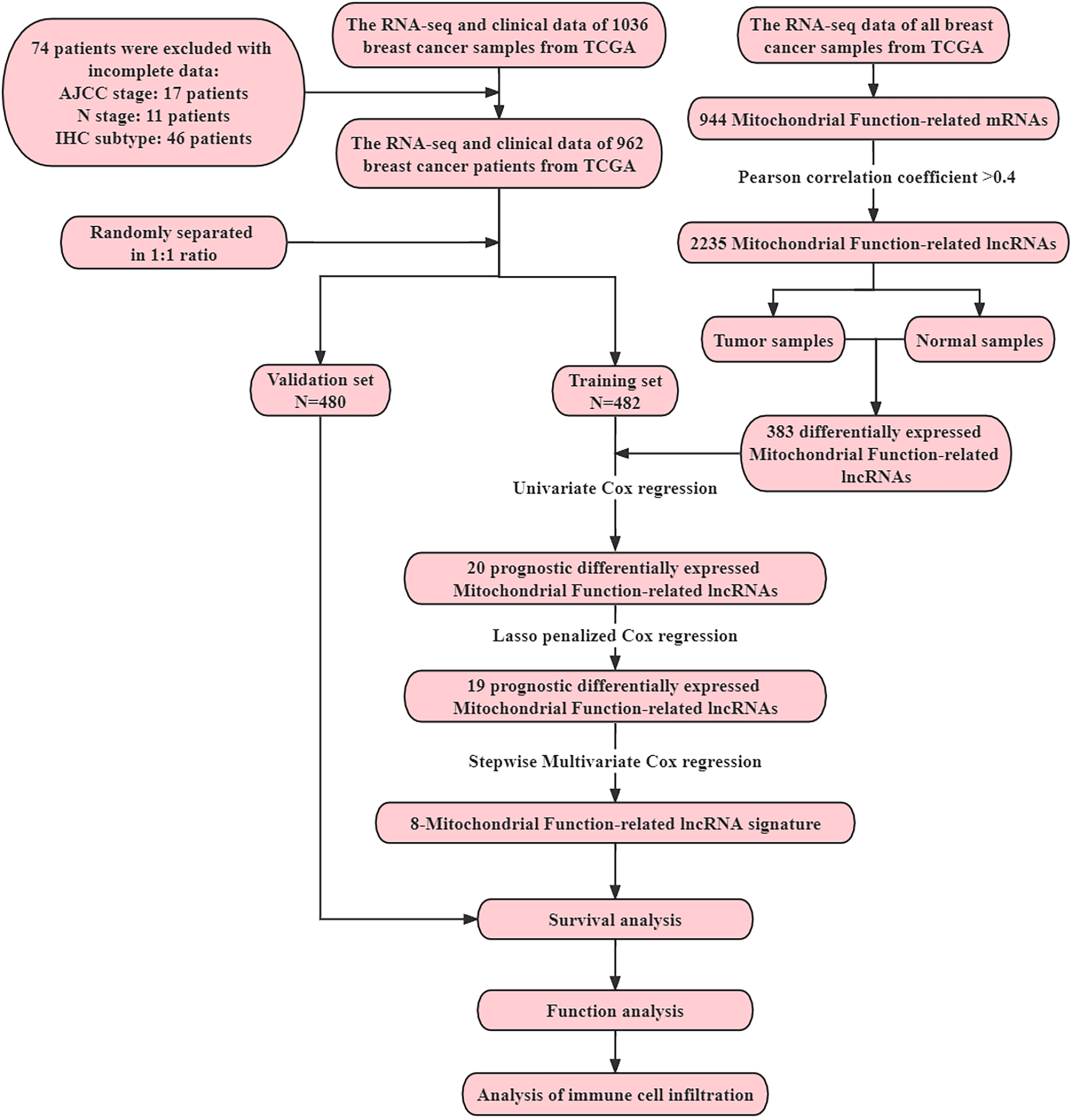
The flow chart of the study.

1036 patients with lobular or ductal BRCA were obtained and further analyzed according to gene expression and corresponding clinical data. The data for estrogen receptor (ER), progesterone receptor (PR), and human epidermal growth factor 2 (HER2) were derived from immunohistochemistry (IHC) and were extracted directly from the clinical information from the TCGA-BRCA database. However, clinical information was missing or inadequate for some samples, a part of the patients presented "unknown" for AJCC stage, T, N, M stage (the 8th edition)^12^, ER, PR, or HER2 status. Patients were excluded if they lacked or did not have sufficient clinical data for analysis. Ultimately, 962 samples were chosen and allocated in a roughly 1:1 ratio to training (n = 482) and validation (n = 480) cohorts using random sampling (Fig. 1).

### Identification of mitochondrial function-related lncRNAs

Firstly, mitochondrial function-related mRNAs were obtained from the MitoMiner 4.0 database^13^, which contained the most exhaustive list of mitochondrial function-related genes. Then, Pearson's correlation coefficients were calculated to define the correlation between the expression of mitochondrial function-related mRNAs and the corresponding lncRNAs and to assess the linkage between them. Mitochondrial function-related lncRNAs were screened by Pearson correlation analysis according to the criteria of P < 0.001 and |r|> 0.4.

### Construction and validation of a prognostic mitochondrial function-related lncRNA signature

The R "limma" package was used to screen out the differentially expressed lncRNAs (DElncRNAs) among tumor and malignant samples from mitochondrial function-related lncRNAs, with the FDR < 0.05 and |log2FC|> 1.0. Then the differentially expressed mitochondrial function-related lncRNAs that were associated with overall survival (OS) in the training cohort were discovered by univariate Cox regression analysis. Subsequently, the candidate lncRNAs were subjected to lasso and stepwise multivariate Cox regression analysis to evaluate their contribution as independent prognostic factors in OS. Finally, these prognostic differentially expressed mitochondrial function-related lncRNAs constituted the prognostic signature.

On the basis of the expression of mitochondrial function-associated lncRNAs in the signature and the accompanying regression coefficients, the risk score of each sample was calculated utilizing the following formula:

$$ \user2{Risk~}\;\user2{Score}\; = \mathop \sum \limits_{{\user2{i} = 1}}^{{\mathbf{n}}} {\mathbf{Coef}}\left( {\mathbf{i}} \right) \times \left( {{\mathbf{expression}}\;~{\mathbf{of}}~\;{\mathbf{lncRNA}}\left( {\mathbf{i}} \right)} \right) $$

Patients in the training cohort were assigned to either lower- or higher-risk clusters based on the median value of the risk score. OS rates were calculated by Kaplan–Meier analysis and compared by log-rank test, and time-dependent ROC curve analysis was performed with the "timeROC" package of R to evaluate the predictive precision of the signature. Then, each sample in the validation cohort has a risk score determined using the same algorithm as the training cohort, and patients in the validation cohort were classified into lower- and higher-risk categories using the same cutoff value as in the training cohort.

### Establishment of the nomogram

Nomogram was created based on risk scores to visualize three- and five-year OS in BRCA, and the accuracy of the prediction of the mitochondrial function-related lncRNA signature was tested using calibration curves.

### Establishment of a lncRNA-mRNA co-expression network

To visualize the relationship between mitochondrial function-related lncRNAs and their associated mRNAs, the co-expression network was depicted using Cytoscape (version 3.9.0). In addition, the correlation between them was shown in the Sankey diagram.

### Gene set enrichment analysis (GSEA)

The molecular and biological disparities among the higher- and lower-risk groups were investigated employing the GSEA (version 4.1.0). The hallmark gene sets (h.all.v7.4.symbols.gmt) were obtained from the Molecular Signatures Database (mSigDB). Genes were sifted utilizing the least and most gene set sizes of 15 and 500, respectively.

### Functional enrichment analysis

Gene Ontology (GO) enrichment analysis^14^ was performed based on the DElncRNAs among the higher- and lower-risk classes to discover the biological processes, molecular functions, and cellular components associated with the mitochondrial function-related lncRNA signature. Meanwhile, the signaling pathways considerably enriched by the signature were determined utilizing the Kyoto Encyclopedia of Genes and Genomes (KEGG) pathway enrichment analysis^15^. GO (c5.go.v7.4.symbols.gmt) and KEGG gene sets (c2.cp.kegg.v7.4.symbols.gmt) were all downloaded from the mSigDB.

### The assessment of relevance to TIM

The CIBERSORT algorithm (version 1.03)^16^ was used to calculate the proportion of each kind of tumor-infiltrating immune cells (TIICs) in samples. The "CIBERSORT" package of R was exploited to compare the abundance of 22 kinds of TIICs in the higher- and lower-risk categories. Besides, we also used the ssGSEA method to calculate the enrichment scores of 28 immune cell types. Additionally, the association between risk types and immune checkpoint molecules, immune functions, and the difference in TIICs was then further analyzed.

### Statistical analysis

Data were processed using Perl (version 5.30.0), and statistical analyses were performed using R (version 4.1.1) or SPSS (version 25.0). To assess the association between the expression levels of mitochondrial function-related mRNAs and associated lncRNAs, the Pearson correlation coefficients were determined, and P < 0.001 was considered significant. In addition, the differences in the proportions of clinical characteristics were examined by the Chi-square test or Mann–Whitney U-test. Lasso penalized Cox regression analysis followed by multivariate Cox regression analysis was performed using the significant prognostic factors identified in the univariate analysis to determine the independent prognostic factors of OS. The Kaplan–Meier method was used to depict survival curves, and the differences were evaluated using the log-rank test. The correlation between TIICs and the risk score was examined by Spearman's correlation analysis. Furthermore, the dissimilarities in the proportions of TIICs, immune checkpoints, and immune functions were compared using the Wilcox test between the higher- and lower-risk categories. The two-sided P value < 0.05 was considered significant.

## Results

### Identification of the differentially expressed mitochondrial function-related LncRNAs in BRCA

**Figure 2:**
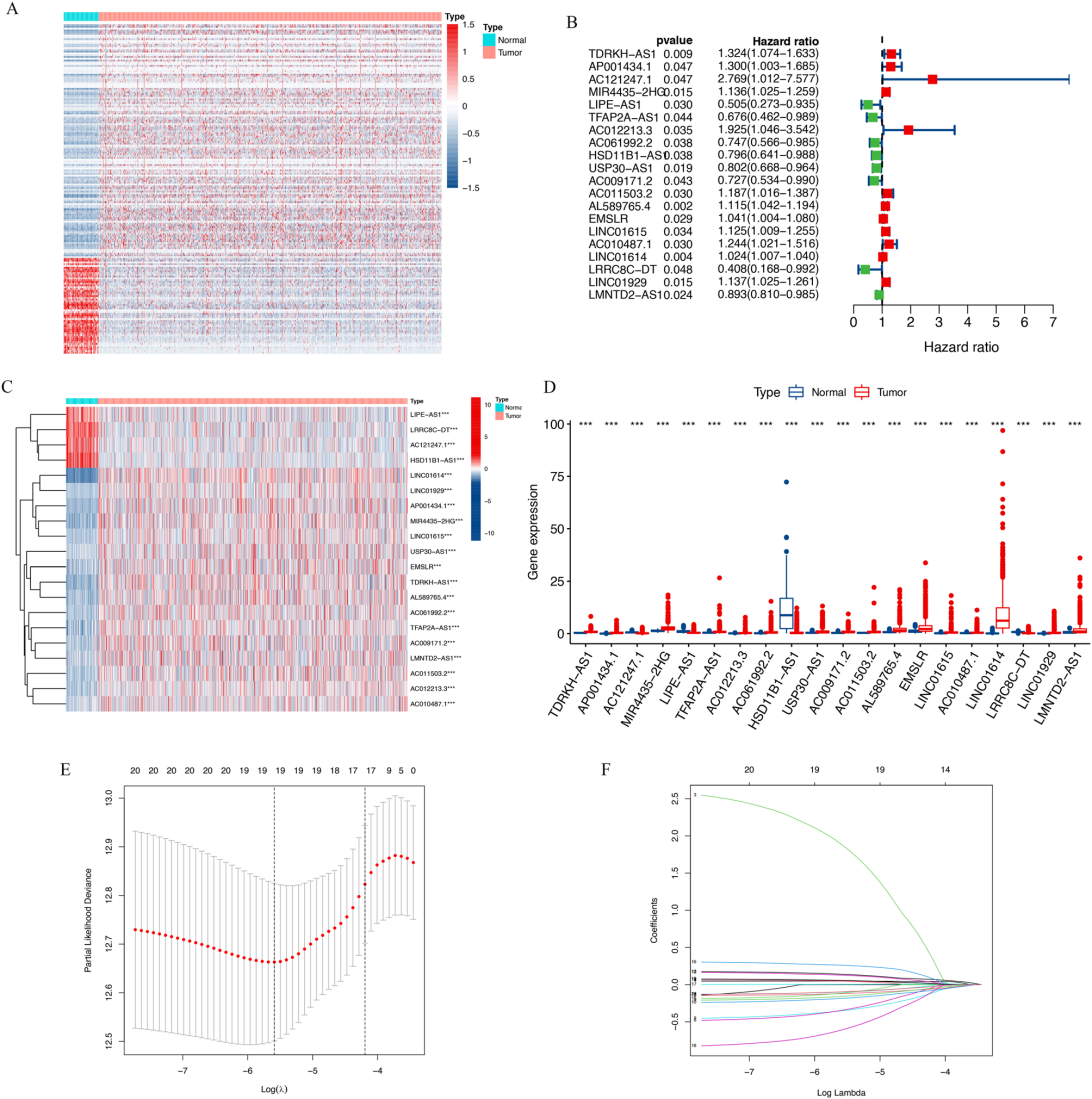
Identification of the prognostic differentially expressed mitochondrial function-related lncRNAs in BRCA patients. (**A**) Heatmap of the 383 differentially expressed mitochondrial function-related lncRNAs. (**B**) Forest plots showing a total of 20 differentially expressed mitochondrial function-related lncRNAs associated with OS in univariate Cox regression analysis. (**C**) Heatmap of the 20 lncRNAs from the results of univariate Cox regression analysis. (**D**) Boxplot of different expressions of the 20 lncRNAs from the results of the univariate Cox regression analysis. (**E**, **F**) The mitochondrial function-related lncRNA signature was then constructed by lasso regression analysis.

Firstly, we obtained 944 mitochondrial function-related mRNAs from the MitoMiner 4.0 database^13^. (Supplement Table S1). Then, according to the coexpression relationship between lncRNAs identified from the RNA-seq data in TCGA-BRCA and the 944 mitochondrial function-related mRNAs, 2235 lncRNAs were recognized as mitochondrial function-related lncRNAs (Supplement Tables S2, S3). Among them, 383 were identified as DElncRNAs. In conclusion, not only were these 383 lncRNAs differentially expressed between normal and tumor samples, but they were also mitochondrial function-related lncRNAs (Fig. 2A, Supplement Table S4).

### Identification of the signature of mitochondrial function-related LncRNAs and construction of a prognostic model in the training cohort

A total of 962 BRCA patients were enrolled in the study with a median follow-up time of 73.333 months (0.003–717.083 months), and they were randomly assigned to either the training group (n = 482) or the validation group (n = 480) (Supplementary Tables S5, S6). Table 1 illustrated that the clinicopathological features of patients in the training and validation groups do not differ statistically significantly.

**Table 1:** Clinical characteristics of patients in training and validation cohorts.

Characteristic		Training cohorts (N = 482)	Validation cohorts (N = 480)	P value
Age	< 60	250 (51.9%)	269 (56.0%)	0.194
	≥ 60	232 (48.1%)	211 (44.0%)	
AJCC stage	Stage I	81 (16.8%)	82 (17.1%)	0.929
	Stage II	282 (58.5%)	275 (57.3%)	
	Stage III	108 (22.4%)	117 (24.4%)	
	Stage IV	11 (2.3%)	6 (1.2%)	
T stage	T1	118 (24.5%)	128 (26.7%)	0.309
	T2	284 (58.9%)	280 (58.3%)	
	T3	61 (12.7%)	64 (13.3%)	
	T4	19 (3.9%)	8 (1.7%)	
N stage	N0	233 (48.3%)	220 (45.8%)	0.438
	N1	163 (33.8%)	167 (34.8%)	
	N2	50 (10.4%)	59 (12.3%)	
	N3	36 (7.5%)	34 (7.1%)	
M stage	M0	471 (97.7%)	474 (98.8%)	0.224
	M1	11 (2.3%)	6 (1.2%)	
ER	Negative	112 (23.2%)	105 (21.9%)	0.626
	Positive	370 (76.8%)	374 (77.9%)	
PR	Negative	167 (34.6%)	146 (30.4%)	0.142
	Positive	311 (64.5%)	333 (69.4%)	
HER2	Negative	354 (73.4%)	349 (72.7%)	0.985
	Positive	90 (18.7%)	89 (18.5%)	
IHC Subtype	Luminal	381 (79.0%)	381 (79.3%)	0.642
	HER2	15 (3.1%)	20 (4.2%)	
	TNBC	75 (1.6%)	70 (1.5%)	

Then we identified the prognostic lncRNAs from these 383 differentially expressed mitochondrial function-related lncRNAs. In the training cohort, the univariate Cox regression analysis revealed that 20 lncRNAs were associated with OS (Fig. 2B–D). Then, lasso regression analysis (Fig. 2E, F) followed by multivariate Cox regression analysis was performed to construct a prognostic model. Eventually, an optimal 8-lncRNA signature (AC121247.1, LIPE-AS1, TFAP2A-AS1, USP30-AS1, AL589765.4, EMSLR, LINC01615, and LRRC8C-DT) was identified based on both stepwise strategy (Table 2). As a result, these 8 lncRNAs constituted the prognostic signature of mitochondrial function-related lncRNAs.

**Table 2:** 8 mitochondrial function-related lncRNAs of the prognostic signature.

Gene symbol	Ensemble ID	Location	Multi-Cox regression coefficient	Uni-Cox regression		
				HR	95% CI	P value
AC121247.1	ENSG00000225399	Chromosome 3: 49,260,085-49,261,316	2.045	2.769	1.012–7.577	0.047
LIPE-AS1	ENSG00000213904	Chromosome 19: 42,397,114-42,652,367	− 0.794	0.505	0.273–0.935	0.030
TFAP2A-AS1	ENSG00000229950	Chromosome 6: 10,409,340-10,416,446	− 0.664	0.676	0.462–0.989	0.044
USP30-AS1	ENSG00000256262	Chromosome 12: 109,052,344-109,053,986	− 0.146	0.802	0.668–0.964	0.019
AL589765.4	ENSG00000249602	Chromosome 1: 151,763,384-151,769,501	0.177	1.115	1.042–1.194	0.002
EMSLR	ENSG00000232445	Chromosome 7: 101,308,270-101,314,800	0.066	1.041	1.004–1.080	0.029
LINC01615	ENSG00000223485	Chromosome 6: 169,157,162-169,163,007	− 0.164	1.125	1.009–1.255	0.034
LRRC8C-DT	ENSG00000231999	Chromosome 1: 89,581,291-89,633,003	− 1.202	0.408	0.168–0.992	0.048

**Figure 3:**
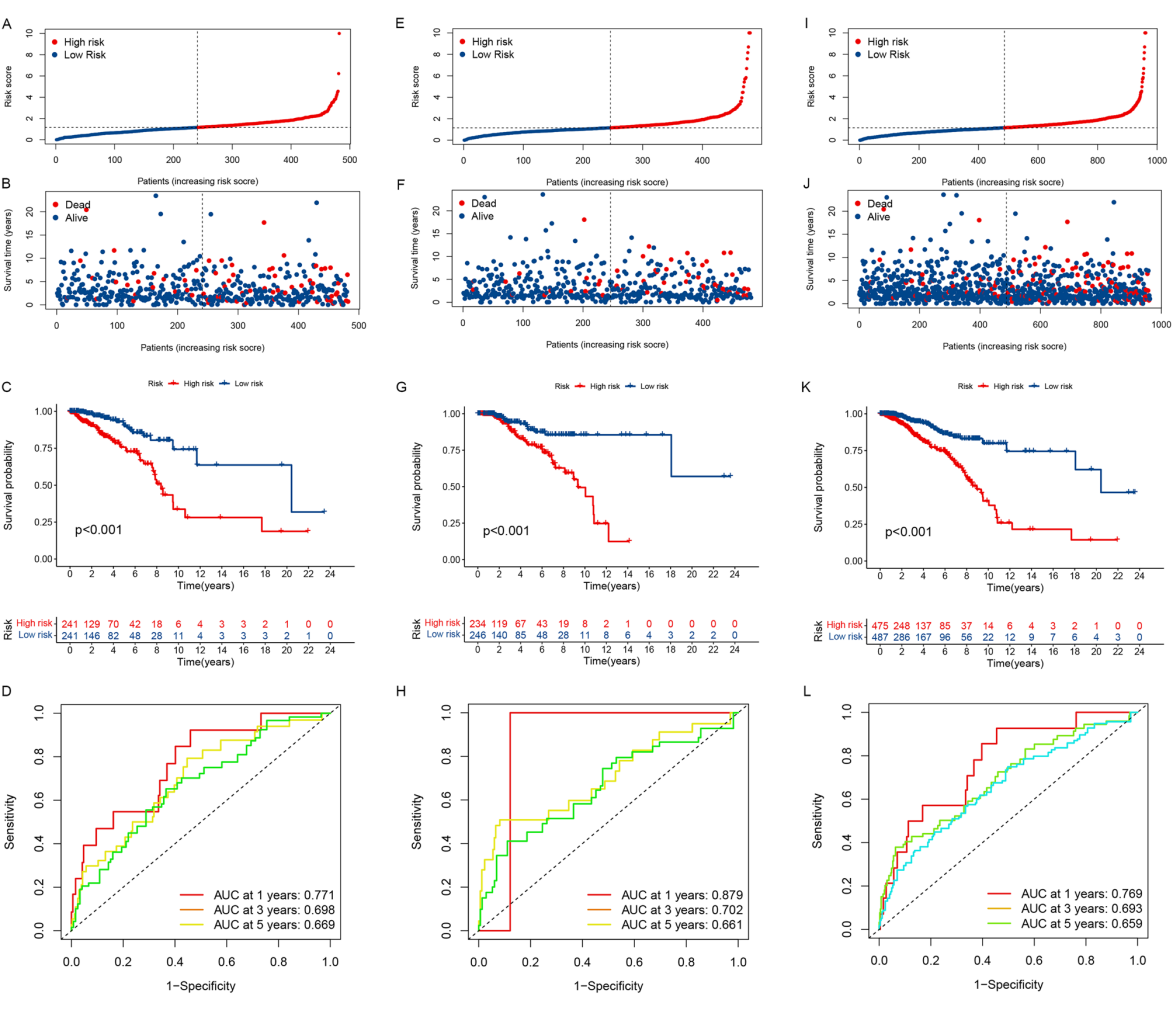
Prognostic analysis of the mitochondrial function-related lncRNA signature model. The distribution of the risk score in the training cohort (**A**), validation cohort (**E**), and entire cohort (**I**). The distributions of overall survival status in the training cohort (**B**), validation cohort (**F**), and entire cohort (**J**). Kaplan–Meier curves for the overall survival of patients in the higher- and lower-risk groups in the training cohort (**C**), validation cohort (**G**), and entire cohort (**K**). AUC of time-dependent ROC curves verified the prognostic accuracy of the risk score in the training cohort (**D**), validation cohort (**H**), and entire cohort (**L**).

**Figure 4:**
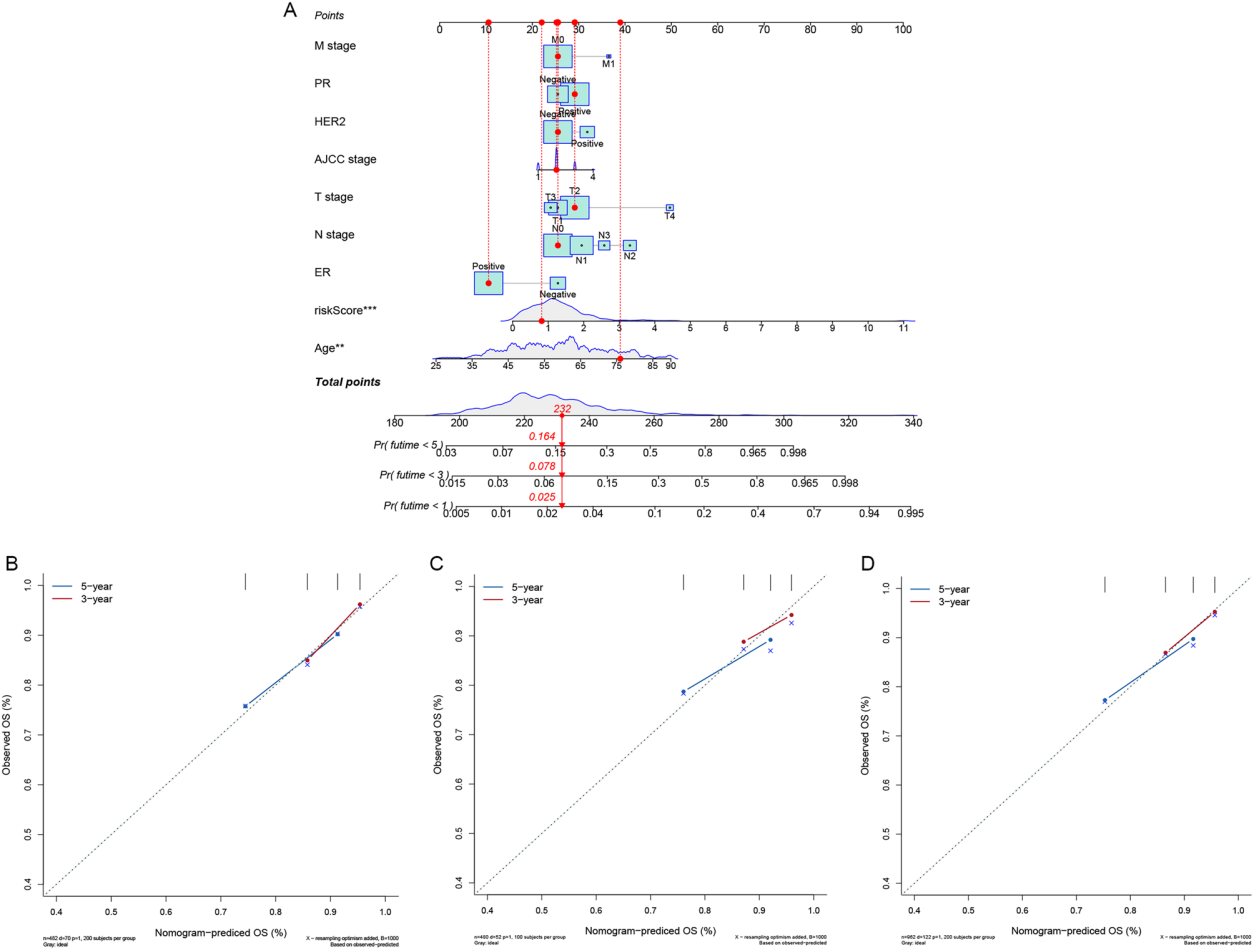
Establishment of the nomogram. (**A**) A nomogram established by integrated traditional clinicopathological indicators and the risk score. Calibration plots for 3- and 5-year OS in the training cohort (**B**), validation cohort (**C**), and entire cohort (**D**).

The patients were classified as higher-risk (n = 241) or lower-risk (n = 241) based on their risk scores (Supplement Table S7). The patients in the higher-risk category had more deaths (Fig. 3A, B). Individuals within the lower-risk category had a better median OS (Fig. 3C). In addition, the ROC curve of the training group indicated that the model had a good predictive value on the OS of BRCA (1-year AUC: 0.771; 3-year AUC: 0.698) (Fig. 3D). Then, the risk score and other clinicopathological indicators such as age, AJCC stage, T, N, M stage, ER, PR, and HER2 status were combined to build a nomogram (Fig. 4A), and the calibration curves revealed that the signature had a high consistency with the actual 3- and 5-year OS (Fig. 4B).

### Validation of the 8-mitochondrial function-related lncRNA signature

Based on the cut-off value identical to that of the training cohort, the individuals in the validation cohort were split into categories of higher-risk (n = 234) and lower-risk (n = 246) (Supplement Table S8). Similarly, the death rate in the higher-risk class was much more elevated in the validation cohort (Fig. 3E, F). The patients in the lower-risk category presented the better OS (Fig. 3G), and the AUC of the ROC curve reached 0.879 in 1 year and 0.702 in 3 years (Fig. 3H), which implied that this 8-mitochondrial function-related lncRNA prognostic signature was efficacious. According to the calibration curve, this prognostic signature exhibited sound agreement between the estimate of the nomogram and the real statement to forecast the periods of OS at 3 and 5 years in the validation cohort (Fig. 4C). In the same way, all findings were presented similarly in the whole cohort (Figs. 3I–L, 4D).

### The independent prognostic significance of the 8-mitochondrial function-related lncRNA signature

**Figure 5:**
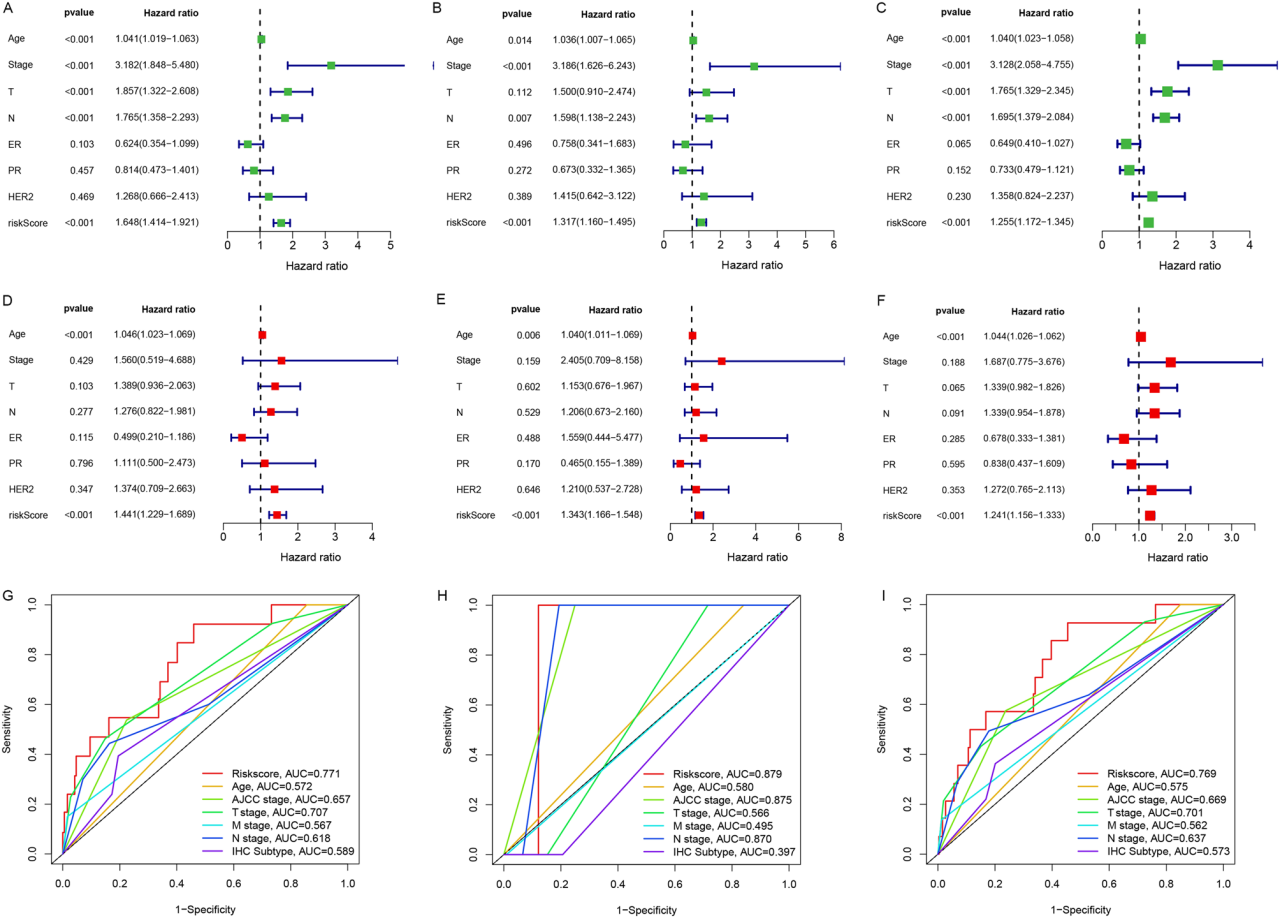
Independent prognostic value of the mitochondrial function-related lncRNA signature. Results of the univariate Cox regression and multivariate Cox regression analysis regarding OS in the training cohort (**A**, **D**), the validation cohort (**B**, **E**), and the entire cohort (**C**, **F**). AUC of ROC curves compared the prognostic accuracy of the risk score and other prognostic factors in the training cohort (**G**), the validation cohort (**H**), and the entire cohort (**I**).

Univariate Cox regression analysis revealed that the risk score was significantly correlated with OS in both the training cohort (HR 1.648, 95% CI 1.414–1.921, P < 0.001, Fig. 5A) and validation cohort (HR 1.317, 95% CI 1.160–1.495, P < 0.001, Fig. 5B). Similarly, which was also remarkably associated with OS in the entire cohort (HR 1.255, 95% CI 1.172–1.345, P < 0.001, Fig. 5C).

In multivariate Cox regression analysis, the risk score is still an independent factor of OS after adjusting for additional variables. (Training cohort: HR 1.441, 95% CI 1.229–1.689, P < 0.001, Fig. 5D; validation cohort: HR 1.343, 95% CI 1.166–1.548, P < 0.001, Fig. 5E; entire cohort: HR 1.241, 95% CI 1.156–1.333, P < 0.001, Fig. 5F).

Furthermore, the AUC values of the prognostic signature were 0.771, 0.879, and 0.769 in the training, validation, and whole cohort, respectively, which were more elevated than the other prognostic clinicopathological indicators (Fig. 5G–I).

### Establishment of the lncRNA-mRNA co-expression network

**Figure 6:**
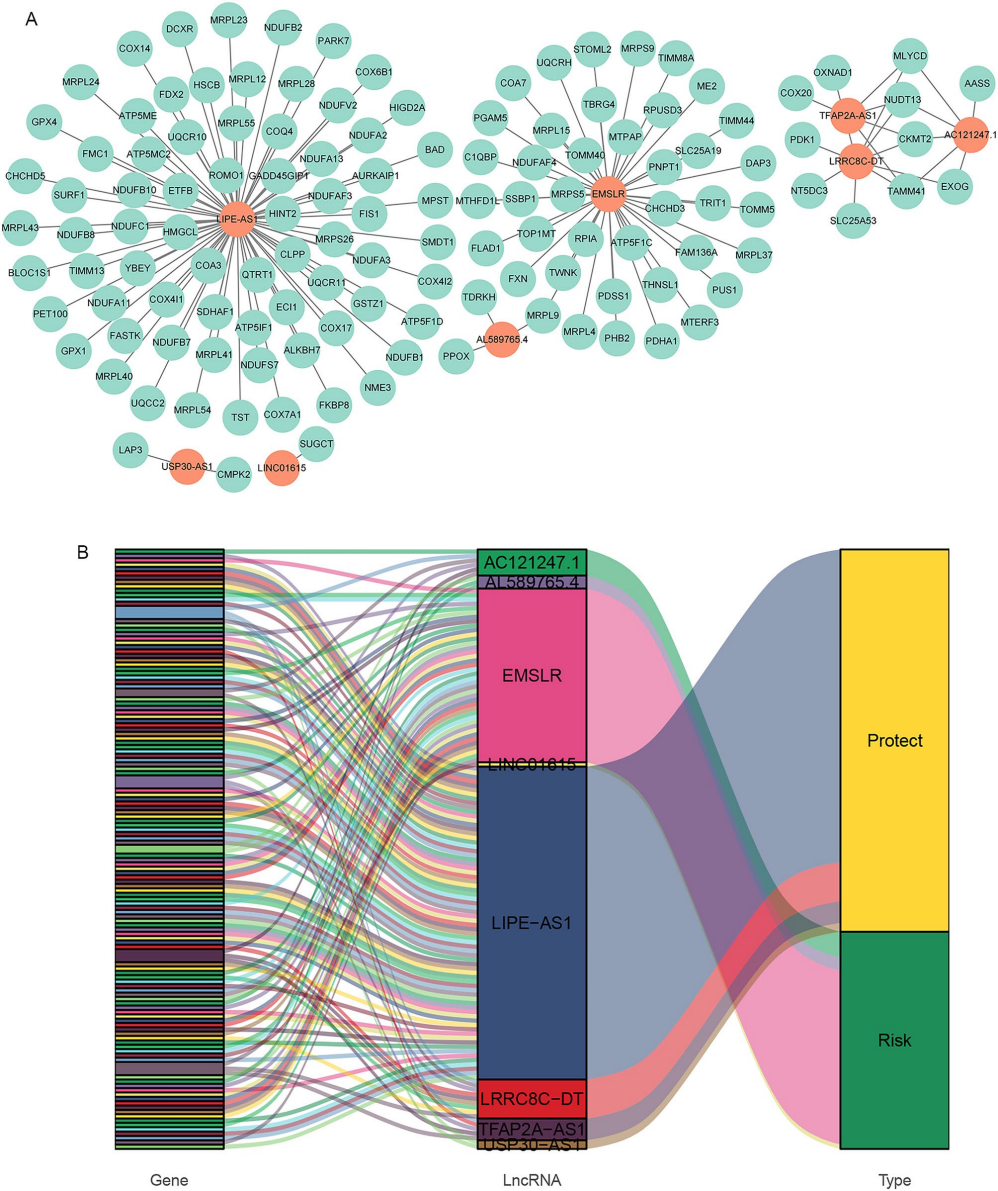
Construction of the mitochondrial function-related lncRNA-mRNA co-expression network. (**A**) Diagram of the mitochondrial function-related lncRNA-mRNA network. (**B**) Sankey diagram shows the connection degree between the mitochondrial function-related lncRNAs and the mitochondrial function-related mRNAs.

The mitochondrial function-related lncRNA signature contained 8 lncRNAs. Using Cytoscape 3.9.0, the lncRNA-mRNA coexpression network was created to demonstrate the prognostic importance of the 8 lncRNAs and their connections to mitochondrial function-associated mRNAs. The network comprised 138 lncRNA-mRNA pairs, as presented in Fig. 6A.

The Sankey diagram depicted not only the link between mitochondrial function-related lncRNAs and mitochondrial function-related mRNAs but also the link between mitochondrial function-related lncRNAs and the risk type of patients with BRCA (Fig. 6B). As shown in Fig. 6B, lncRNA LIPE-AS1 had a co-expression relationship with 72 mitochondrial function-related mRNAs, and there was a co-expression relationship between lncRNA EMSLR and 40 mitochondrial function-related mRNAs (Supplement Table S9). Furthermore, we observed that LIPE-AS1, TFAP2A-AS1, USP30-AS1, and LRRC8C-DT functioned as protective factors with HR < 1, and AC121247.1, AL589765.4, LINC01615, and EMSLR acted as risk factors with the HR > 1 (Fig. 6B, Table 2).

### Discovery of important hallmarks and functional analysis

**Figure 7:**
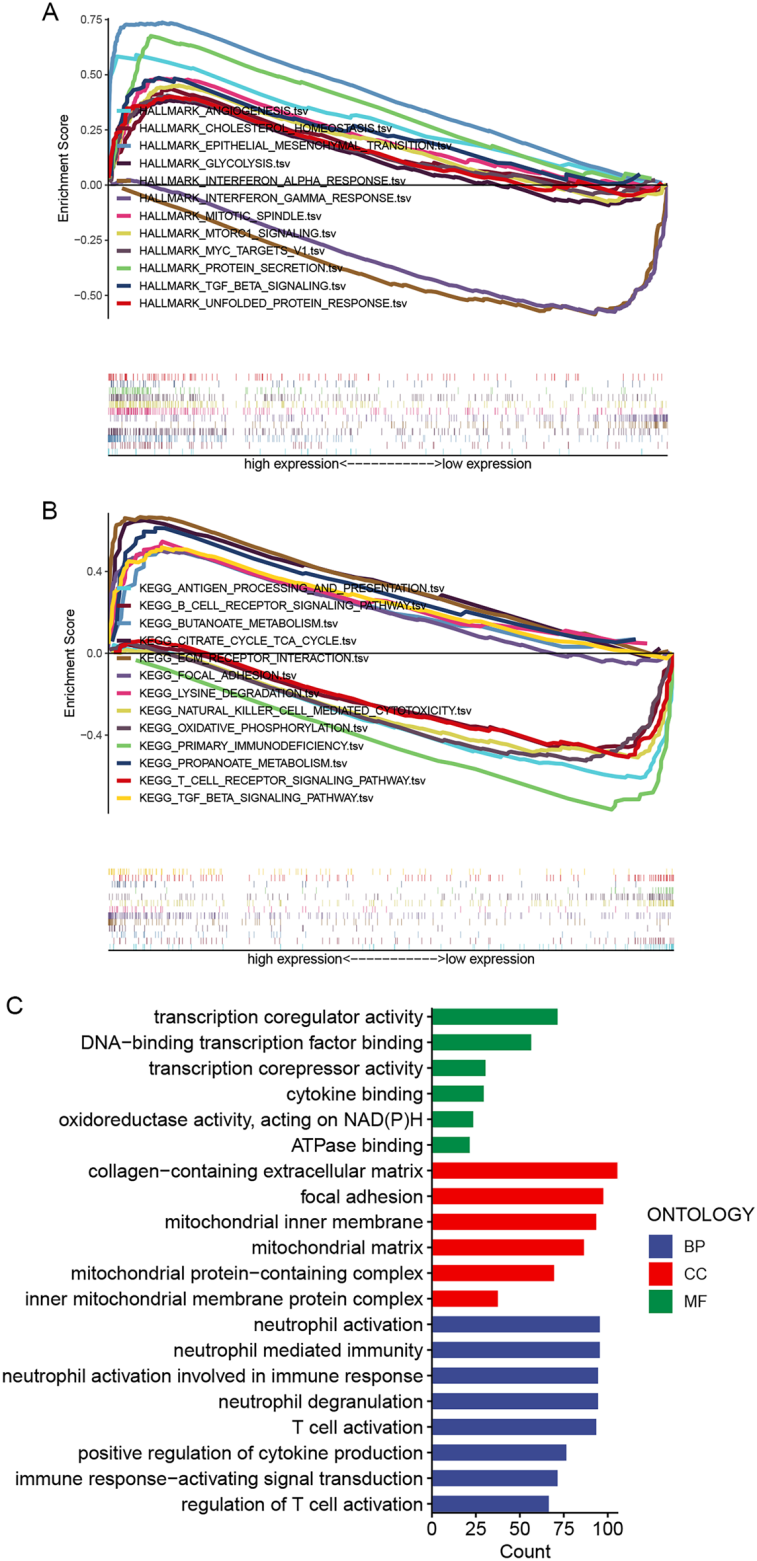
Functional enrichment analysis of higher-risk and lower-risk groups based on the mitochondrial function-related lncRNA prognostic signature. (**A**) GSEA results show significant enrichment of metabolic-related pathways in higher-risk BRCA patients. (**B**) KEGG results show significant enrichment of metabolic-related pathways in the higher-risk group, and the immune-related pathways were activated in the lower-risk BRCA patients. (**C**) GO results show the DElncRNAs between higher and lower-risk groups majorly enriched in the mitochondrial function-related and immunoregulatory-related functions and pathways.

To investigate the signal transduction pathways and biological functions, we performed the GSEA of DElncRNAs between the higher- and lower-risk categories. The results demonstrated that functional annotation, with more metabolic-related hallmarks such as glycolysis, cholesterol homeostasis, and the TGF-beta signaling pathway was up-regulated in the higher-risk patients. Meanwhile, both the interferon-alpha and interferon-gamma response signaling pathways were upregulated in the lower-risk category (Fig. 7A, Supplement Fig. S1).

GO enrichment and KEGG pathway analysis were performed to further explore the biological functions of these lncRNAs. The KEGG pathway analysis revealed that the DElncRNAs were enriched in metabolic-related pathways in the higher-risk category, including the citrate cycle TCA cycle, butanoate metabolism, propanoate metabolism, and the TGF-beta signaling pathway. Meanwhile, the immune-related pathways such as antigen processing and presentation, B cell receptor signaling pathway, natural killer cell-mediated cytotoxicity, T cell receptor signaling pathway, and primary immunodeficiency were activated in the lower-risk category (Fig. 7B, Supplement Fig. S2). GO enrichment analysis indicated that DElncRNAs were remarkably enriched in mitochondrial function-related cellular components, such as mitochondrial inner membrane, mitochondrial matrix, mitochondrial protein-containing complex, and inner mitochondrial membrane protein complex. In terms of molecular functions, they were enriched in transcription coregulator activity, DNA-binding transcription factor binding, cytokine binding, oxidoreductase activity, acting on NAD(P)H, and ATPase binding. Meanwhile, immunoregulatory-related biological processes were also enriched significantly, such as the biological processes of neutrophil activation, neutrophil-mediated immunity, neutrophil activation involved in immune response, neutrophil degranulation, T cell activation, positive regulation of cytokine production, immune response-activating signal transduction, and regulation of T cell activation (Fig. 7C).

In conclusion, the above results suggest that this mitochondrial function-related lncRNA signature may not only be related to mitochondrial metabolism, but also to TIM.

### The immune cells infiltration landscape in BRCA

**Figure 8:**
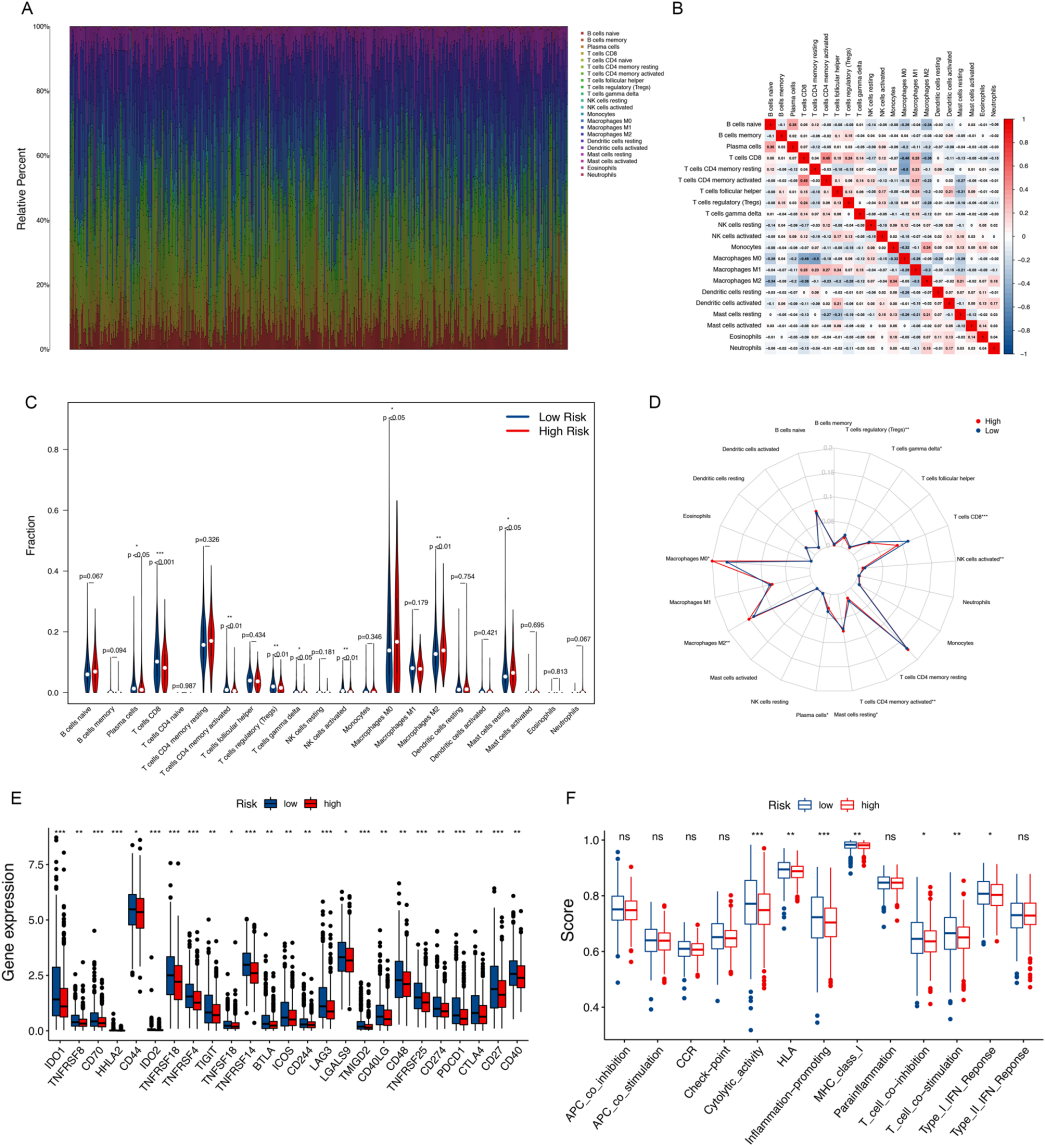
The immune cell infiltration landscape in BRCA. (**A**) Barplot of the proportions of the tumor-infiltrating cell. (**B**) Correlation matrix of the proportions of the tumor-infiltrating cell. (**C**) Violin plot showed the different proportions of tumor-infiltrating cells between the higher-risk and lower-risk groups. (**D**) Radar plot showed the different proportions of tumor-infiltrating cells between the higher-risk and lower-risk groups. (**E**) The expression levels of immune checkpoint molecules in the higher-risk and lower-risk groups. (**F**) The scores of immune functions in the higher-risk and lower-risk groups.

Given the result that DElncRNAs are enriched in immune-related functions and pathways, to further understand the link between the signature and tumor immunity, we investigated the case of TIICs, and we further investigated the relevance of the prognostic mitochondrial function-related lncRNA signature to the TIM of the BRCA. The proportion of TIICs in each sample of the training cohort was illustrated in a barplot (Fig. 8A), and Fig. 8B exhibits the fraction of all types of TIICs in a correlation matrix.

Among all types of TIICs, the macrophages M0, M2, and mast cells resting were positively associated with the risk score, while T cells CD8, T cells CD4 memory activated, T cells regulatory (Tregs), T cells gamma delta, NK cells activated, and plasma cells demonstrated the negative correlations (Supplement Fig. S3). To compare the differences in TIICs between the higher- and lower-risk categories, we constructed the plots showing that the abundances of the macrophages M0 and M2 in the higher-risk category were significantly more elevated than those of the lower-risk category, which were the TIICs facilitated tumor proliferation or metastasis^17–20^. While the proportions of infiltrated tumor-killing immune cells, such as T cells CD8, T cells CD4 memory activated, T cells regulatory (Tregs), T cells gamma delta, NK cells activated, and plasma cells in the lower-risk category were much higher, which play crucial roles in the progression of BRCA and function as the tumor suppressors^21–23^ (Fig. 8C, D). The increased infiltration of tumor-killing immune cells suggested that patients in the lower-risk category tended to be the immunologically "hot" tumor.

Then we looked into the degrees of immune checkpoint genes and discovered that 25 different types of them were more elevated in the lower-risk class, including immunosuppressor molecules such as PDCD1, CTLA4, BTLA, LAG3, and TIGIT (Fig. 8E). In addition, we also compared the immune functions, and the results demonstrated that the functions of cytolytic activity, HLA, inflammation-promoting, MHC class I, T cell co-inhibition, T cell co-stimulation, and type I IFN response in the lower-risk category were all higher than those in the higher-risk category (Fig. 8F).

Also, the results of ssGSEA were similar to those of the CIBERSORT method. The ssGSEA analysis showed that the CD8+ T cells, B cells, Th1 cells, TIL, and NK cells in the higher-risk category were significantly lower, and macrophages were more elevated in the higher-risk category. Besides, also similar is that the value of the cytolytic activity, HLA, inflammation-promoting, MHC class I, T cell co-inhibition, T cell co-stimulation, and type I IFN response were all higher in the lower-risk category (Supplement Fig. S4).

### Correlation between mitochondrial function-related lncRNA signature and TIM in Luminal and non-luminal subtypes of BRCA

**Figure 9:**
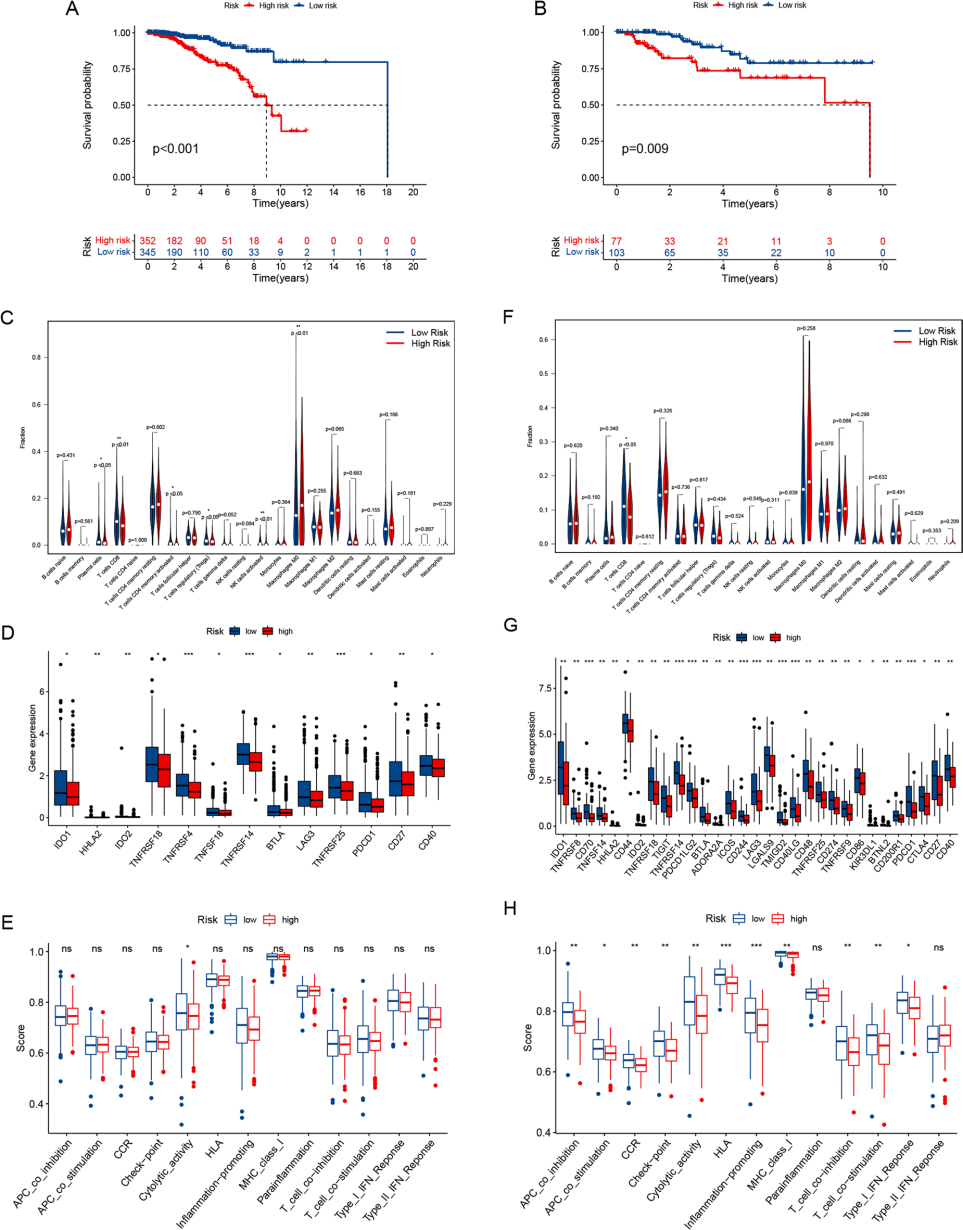
The overall survival and immune cell infiltration landscape in luminal or non-luminal subtypes of BRCA. Kaplan–Meier curves for the overall survival of patients in the higher- and lower-risk groups in luminal BRCA (**A**) and non-luminal BRCA (**B**). The violin plot showed the different proportions of tumor-infiltrating cells between higher-risk and lower-risk groups in luminal BRCA (**C**) and non-luminal BRCA (**F**). The expression levels of immune checkpoint molecules in the higher-risk and lower-risk groups in luminal BRCA (**D**) and non-luminal BRCA (**G**). The scores of immune functions in the higher-risk and lower-risk groups in luminal BRCA (**E**) and non-luminal BRCA (**H**).

The role of the mitochondrial function-related lncRNA signature was investigated in both luminal and non-luminal subtypes of BRCA. The risk score based on the prognostic signature was significantly correlated with OS in patients with luminal or non-luminal BRCA, as shown in Fig. 9A, B, and patients with a heightened risk score had a shorter survival time.

In both luminal and non-luminal BRCA subtypes, we looked at the link between risk score and TIM. The percentage of macrophage M0 in the higher-risk category was considerably higher in the luminal subtype. Plasma cells, CD8 T cells, CD4 memory-activated T cells, regulatory T cells (Tregs), and activated NK cells decreased substantially in the higher-risk category (Fig. 9C). Immune checkpoints were investigated and 13 types of immune checkpoint molecules, such as PDCD1, BTLA, and LAG3, were found to increase in the lower-risk category in the luminal subtype of BRCA (Fig. 9D). Furthermore, we looked at immunological functions and discovered that the lower-risk category had increased cytolytic activity (Fig. 9E).

A similar trend was found in the non-luminal type of BRCA. Firstly, the level of T cell CD8 was more elevated in the lower-risk patients (Fig. 9F). Next, in terms of the immune checkpoints, a total of 31 kinds of immune checkpoint molecules, such as PDCD1, CTLA4, BTLA, LAG3, and TIGIT, were immunosuppressor molecules and relatively higher in the lower-risk patients (Fig. 9G). Furthermore, we compared immune functions in non-luminal BRCA and discovered that the lower-risk category had higher levels of APC co-inhibition, APC co-stimulation, CCR, check-point, cytolytic activity, and HLA, inflammation-promoting, MHC class I, T cell co-inhibition, T cell co-stimulation, and type I IFN response (Fig. 9H).

The above findings showed that the mitochondrial function-related lncRNA signature held a consistent role in both luminal and non-luminal subtypes of BRCA. These outcomes demonstrated a link between the prognostic signature and the TIM in different subtypes of BRCA, indicating that the mitochondrial function-related lncRNA signature may make a difference in immune regulation.

## Discussion

The establishment of targets based on the molecular biological characteristics of tumors has become the key to individualized precision treatment of BRCA. With the expansion of the human genome project and the advent of the post-genomic era, increasing numbers of noncoding RNAs (ncRNAs) were discovered. More than 90% of the transcript products of the human genome are ncRNAs, which are RNAs that do not participate in protein coding but are involved in the regulation of gene expression at different levels^24^. Among them, lncRNAs are involved in crucial physiological processes, such as metabolism and immunity, and are closely related to the occurrence and development of BRCA^25^. At the same time, lncRNAs also participate in regulating mitochondrial function^26^.

Mitochondrial dysfunction can result in cancer-like characteristics, and tumor progression is a metabolic disorder characterized by abnormal energy balance and mitochondrial dysfunction^27,28^. As a consistent characteristic of a wide range of malignancies, mitochondrial dysfunction is primarily responsible for dysregulated energy metabolism in tumor cells and has been considered a trademark of cancer^29–34^. Mitochondrial function-related lncRNAs, including RNA produced from mtDNA as well as nuclear-encoded lncRNAs carried into mitochondria, can regulate mitochondrial gene expression and function^34,35^. Additionally, mitochondrial function-related lncRNAs are critical components of many gene regulatory networks, with the potential to serve as epigenetic messengers to correspond to nuclear and mitochondrial processes^36,37^. Moreover, oncogenesis could be triggered by abnormal regulation of mitochondrial function-related lncRNAs^38^.

With the development of high-throughput sequencing technology, more and more lncRNAs have been confirmed to play an important role in the development of BRCA, making them a promising new therapeutic target for BRCA. And the research between lncRNA and BRCA has gradually become a hot topic in recent years. LncRNAs can be involved in regulating mitochondrial function and influence tumor progression. Mitochondrial LncRNA GAS5 plays a key role in regulating the mechanism and progression of BRCA cells^26^. LncRNA UCA1 improved mitochondrial function for bladder cancer cells and may operate as a probable target for diagnosis and treatment^39^. Besides, lncRNA MALAT1 controls metabolic reprogramming and regulates mitochondrial function in hepatoma carcinoma cells^40^.

However, limited studies are focused on mitochondrial function-associated lncRNAs in BRCA. It has not been reported much in BRCA due to a lot of research still being in its infancy. Therefore, we focused on the relationship between mitochondrial function-related lncRNAs and BRCA. Using bioinformatics and statistical techniques, we comprehensively examined the precision of mitochondrial function-related lncRNAs in the prognostic forecast in BRCA. To begin with, 20 mitochondrial function-related lncRNAs that remarkably correlated with OS were identified by univariate regression analysis. Then, 8 mitochondrial function-related lncRNAs (AC121247.1, LIPE-AS1, TFAP2A-AS1, USP30-AS1, AL589765.4, EMSLR, LINC01615, and LRRC8C-DT) were selected to construct a prognostic signature based on their contribution in multivariate Cox regression.

Next, the risk score of each sample was calculated according to the signature. Patients in the higher-risk group had a shorter OS. What is more, the ROC curves confirmed the accurateness of this signature, which is more reliable than the other conventional clinicopathological indicators in the prediction of the outcome. Furthermore, robust nomograms that predicted 3- and 5-year OS rates were constructed to prove the survival prediction capacity. Meanwhile, calibration plots revealed that the nomograms matched the actual condition. Overall, the mitochondrial function-related lncRNA signature could precisely forecast OS and exhibits considerable potential for practical applications of personalized outcomes for patients with BRCA.

Mitochondria operate as a critical center in the signaling system that controls both natural and adaptive immunity^41^. Mitochondrial dysfunction participates in multiple procedures closely related to the abnormity of the immune system^9^. Considerable research in recent years has realized that there may be certain connections between mitochondria and immunity. Mitochondria are involved in the immunological response, and changeovers in mitochondrial function have been found linked to defective immunity^42^. However, the involvement of mitochondrial function, particularly mitochondrial function-related lncRNAs, in the TIM of BRCA is yet unknown. Our study found that metabolic and mitochondrial function-related cellular components were markedly enriched in the functional enrichment analysis. Interestingly, immune-related biological processes, such as neutrophil activation, neutrophil-mediated immunity, neutrophil activation involved in immune response, neutrophil degranulation, T cell activation, immune response-activating signal transduction, and regulation of T cell activation were also considerably enriched in the functional enrichment analysis of the DElncRNAs between higher- and lower-risk categories. In addition, we also discovered significant activation of immune-related pathways in the lower-risk group, such as antigen processing and presentation, B cell receptor signaling pathway, natural killer cell-mediated cytotoxicity, and T cell receptor signaling pathway. Therefore, we may reasonably conclude that mitochondrial function is closely associated with tumor immunity in BRCA. Current investigation revealed that mitochondrial function is necessary for antigen presentation and processing^43–45^. Buck et al. found that the effector T lymphocytes are marked by owning fused mitochondria^46^. Quintana et al. also discovered that mitochondria are collected in the immune synapse of T lymphocytes^47^. These findings suggest that mitochondrial function is closely associated with tumor immunity, which is consistent with our speculation.

Given that DElncRNAs are enriched in immune-related functions and pathways, to further understand the link between the prognostic mitochondrial function-related lncRNA signature and tumor immunity, we investigated the case of TIICs, and we further investigated the relevance of the signature to TIM of BRCA. We found that the infiltrated tumor-killing immune cells in the higher-risk category were significantly lower, such as T cells CD8, T cells CD4 memory activated, and NK cells activated, which function as tumor suppressors in the progression of malignancy. While the TIICs that accelerated tumor proliferation and metastasis, such as macrophages M0 and M2, which make a vast difference in cancer progression, and participate in tumor invasion, metastasis, and immunosuppression^19^, were significantly more elevated in the higher-risk category. In addition, the results of ssGSEA were similar to those of the CIBERSORT method. The ssGSEA analysis showed that the CD8+ T cells, B cells, Th1 cells, TIL, and NK cells in the higher-risk category were significantly lower, and macrophages were more elevated in the higher-risk category. Besides, also similar is that the value of the cytolytic activity, HLA, inflammation-promoting, MHC class I, T cell co-inhibition, T cell co-stimulation, and type I IFN response were all higher in the lower-risk category. Both the CIBERSORT and the ssGSEA method indicated that mitochondrial function is highly connected with the abundance of TIICs in BRCA and that patients in the higher-risk category have a relatively lower infiltration of tumor-killing immune cells. Furthermore, based on this signature, BRCA patients in the higher-risk category had relatively reduced levels of immune checkpoint molecules and immune functions.

ICIs therapy has made great breakthroughs in immunotherapy for BRCA. In patients with solid tumors, ICIs responders exhibit a "hot" phenotype marked by T lymphocyte infiltration, while non-responders may exhibit a "cold" phenotype characterized by the absence or lack of T cells in the tumor parenchyma^48^. Most "immunologically cold" solid tumors are not responsive to ICIs therapy, and efficacy is often poor. However, it has been proven that most BRCA is known as the "cold" tumor, and the efficacy of ICIs in BRCA is limited^49^. In our study, the infiltration of tumor-killing immune cells was significantly decreased in the higher-risk category of samples. Combining the above theories and the results of our analysis, we believe that further prospective and retrospective studies could be worthwhile to explore the differences in efficacy between high- and low-risk patients for ICI treatment.

Although our analysis utilizes representative and robust data derived from the TCGA public databases, there are still some limitations. Firstly, it is necessary to confirm the importance of the signature by independent validation. Besides, further verification of the signature by the construction of a local cohort is needed in future studies. In addition, multicenter examinations and further prospective analysis or retrospectively with more robust data sets were needed to verify the role of this prognostic signature that we put forward. Finally, since our study preliminarily discovered the association between mitochondrial function related-lncRNAs and tumor immunity, underlying and potential mechanisms need to be further investigated by experimentation in the future.

## Conclusion

In conclusion, we identified the mitochondrial function-related lncRNAs associated with the prognosis of BRCA and developed a prognostic model. Besides, the mitochondrial function risk score was linked to TIICs as well as the levels of immune checkpoint molecules and immune functions. Consequently, the mitochondrial function-related lncRNA signature may have potential significance in the prognosis and serve as a therapeutic target for BRCA.

## Supplementary Information

Supplementary Information.

## Data Availability

The datasets analyzed during the current study are available in the TCGA repository (https://cancergenome.nih.gov/). The original contributions presented in the study are included in the article/Supplementary Materials. Further inquiries can be directed to the corresponding author.
