# Supplementary material for: A mitochondrial function-related LncRNA signature predicts prognosis and immune microenvironment for breast cancer
Source: Sci Rep. 2023 Mar 8;13:3918. doi: 10.1038/s41598-023-30927-y (PMC9995529; doi:10.1038/s41598-023-30927-y)
Supplement: Supplementary file 1 — Supplementary Information. [file 41598_2023_30927_MOESM1_ESM.zip › Supplementary Materials/Supplementary Materials legends.docx]

Table S1: The list of 944 mitochondrial function-related mRNAs obtained from the MitoMiner 4.0 database.

Table S2: The coexpression relationship between the lncRNAs and mitochondrial function-related mRNAs.

Table S3: Expression of the 2235 mitochondrial function-related lncRNAs in breast cancers.

Table S4: The differential expression of the 383 mitochondrial function-related lncRNAs between tumor samples and normal samples.

Table S5: 482 patients were assigned to the training group (n=482).

Table S6: 480 patients were assigned to the validation group (n=480).

Table S7: The patients in the training group were divided into higher- and lower-risk groups according to the median risk score as the cut-off value.

Table S8: The patients in the validation group were divided into higher- and lower-risk groups according to the median risk score as the cut-off value.

Table S9: The relationship between mitochondrial function-related lncRNAs and mitochondrial function-related mRNAs.

Figure S1: GSEA results of differentially expressed genes between higher- and lower-risk groups.

Figure S2: KEGG results of differentially expressed genes between higher- and lower-risk groups.

Figure S3: The correlation between tumor-infiltrated immune cells and the risk score.

Figure S4: The analysis of immune cell infiltration by ssGSEA.
