# Supplementary figures and images for: A mitochondrial function-related LncRNA signature predicts prognosis and immune microenvironment for breast cancer
Source: Sci Rep. 2023 Mar 8;13:3918. doi: 10.1038/s41598-023-30927-y (PMC9995529; doi:10.1038/s41598-023-30927-y)

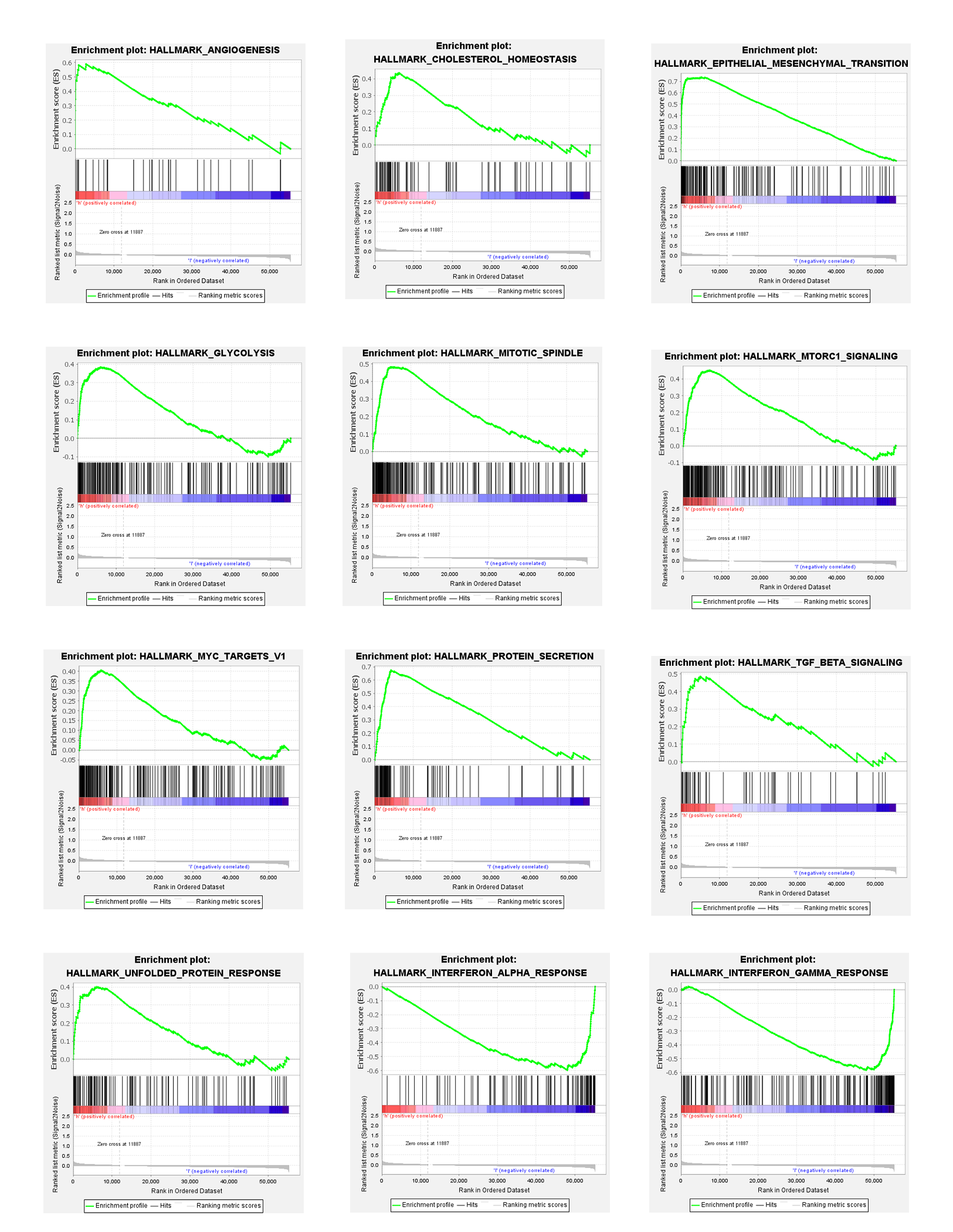

Supplement: Supplementary file 1 — Supplementary Information. [file 41598_2023_30927_MOESM1_ESM.zip › Supplementary Materials/Figure S1.tif]

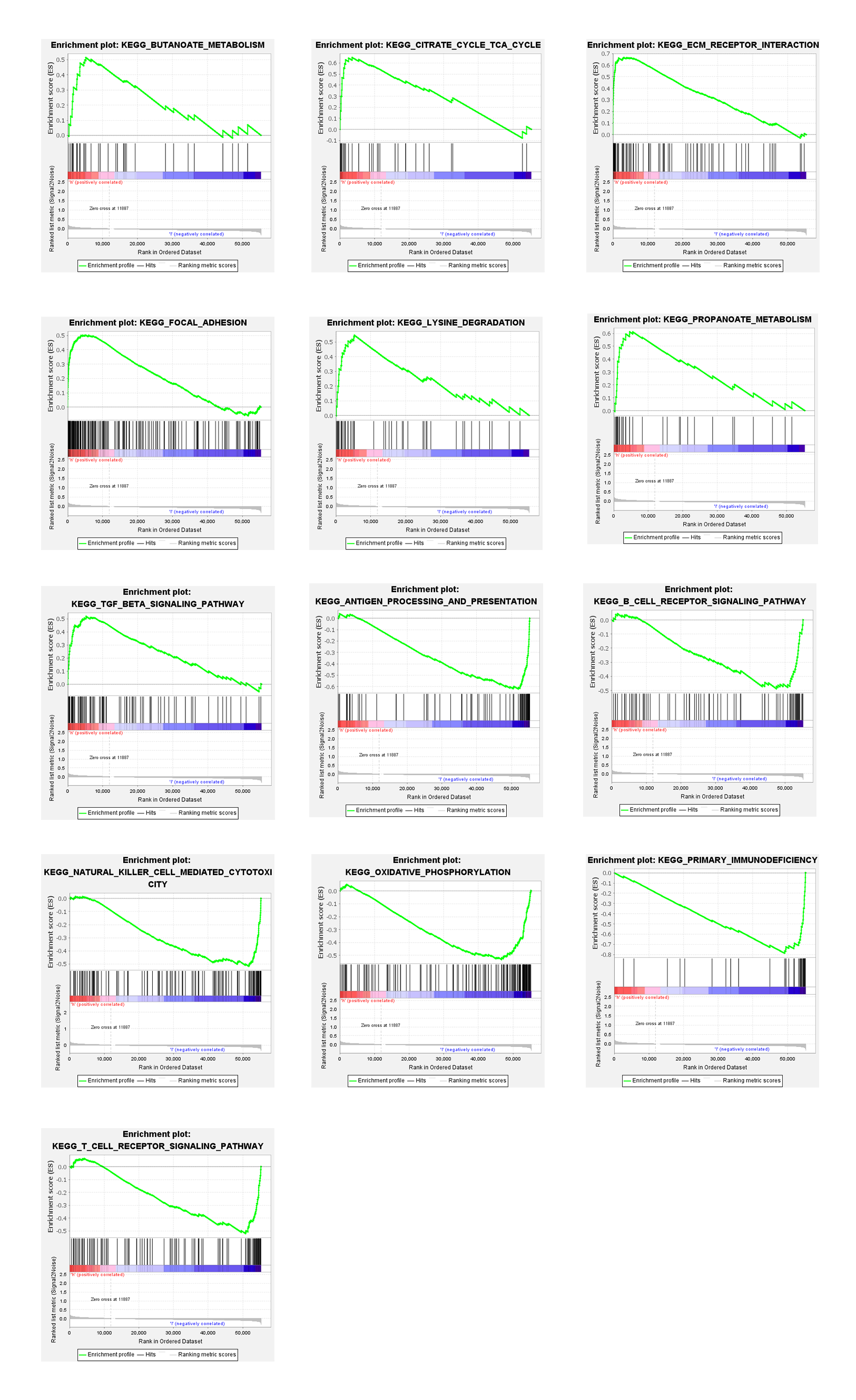

Supplement: Supplementary file 1 — Supplementary Information. [file 41598_2023_30927_MOESM1_ESM.zip › Supplementary Materials/Figure S2.tif]

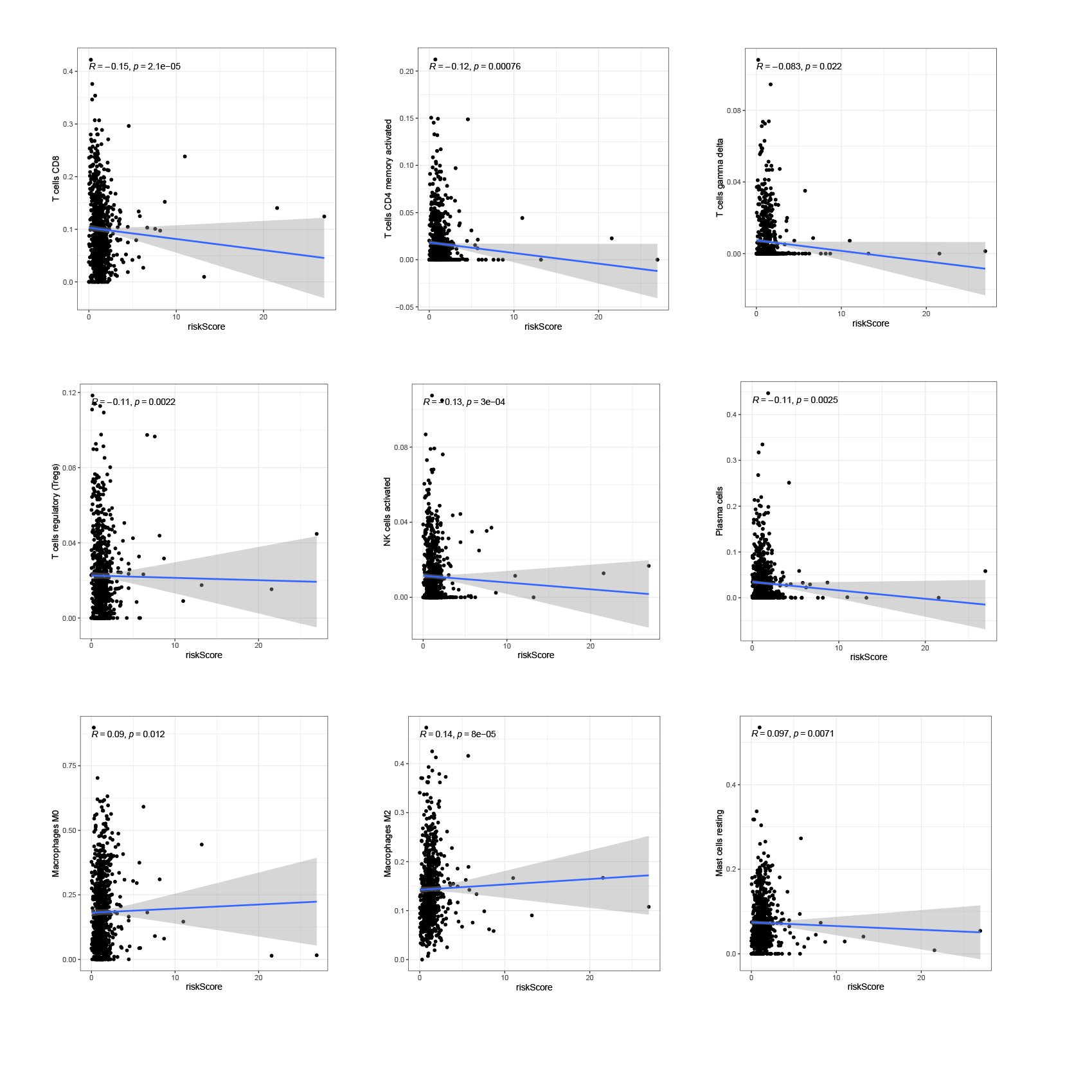

Supplement: Supplementary file 1 — Supplementary Information. [file 41598_2023_30927_MOESM1_ESM.zip › Supplementary Materials/Figure S3.tif]

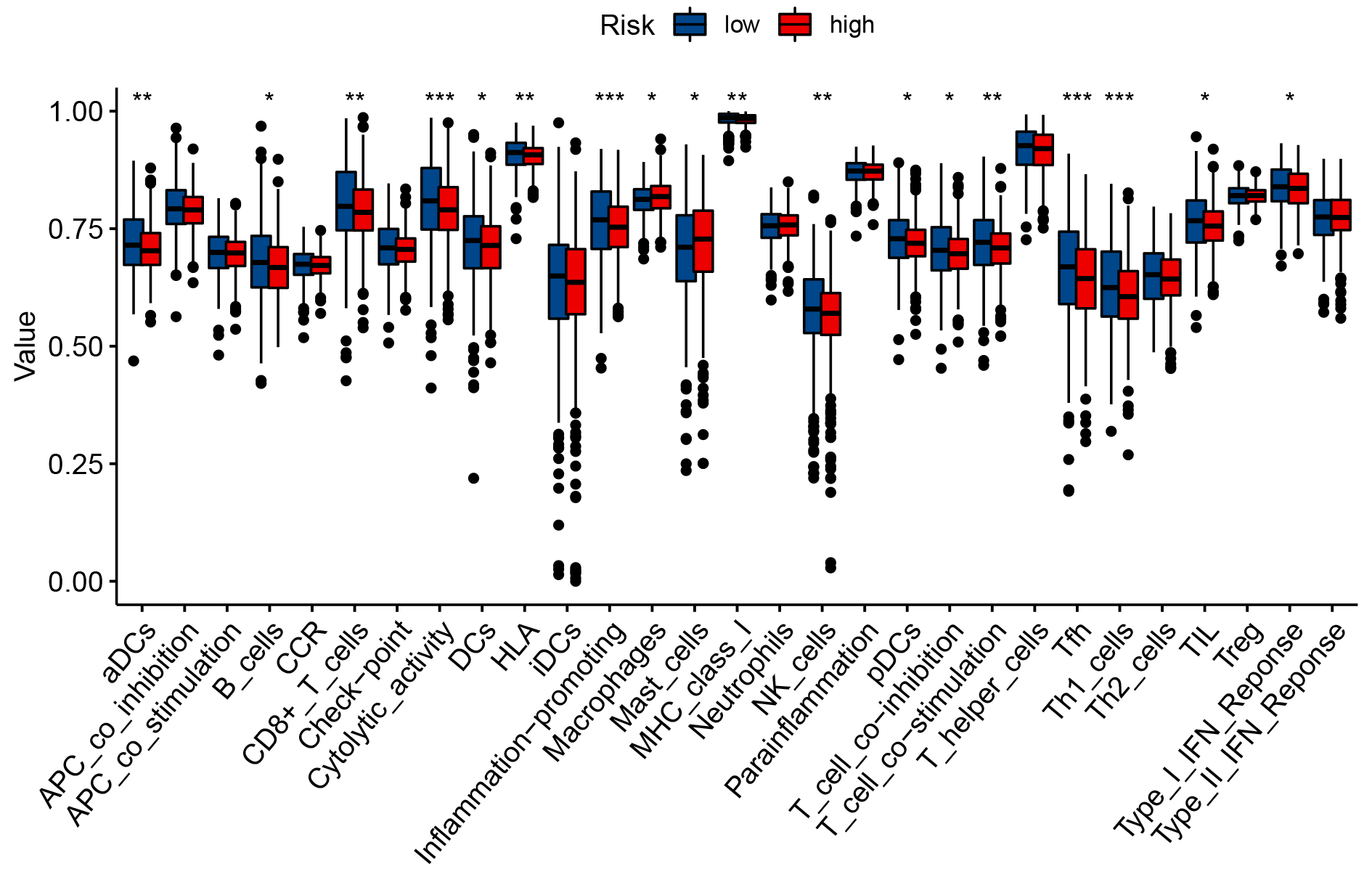

Supplement: Supplementary file 1 — Supplementary Information. [file 41598_2023_30927_MOESM1_ESM.zip › Supplementary Materials/Figure S4.tif]
